# Retrospect of the Medical Sciences

**Published:** 1843-04-01

**Authors:** 


### RETROSPECT OF THE MEDICAL SCIENCES.

### TREATMENT OF VESICO-VAGINAL FISTULA.

Dr. Reid read a paper on the vesico-vaginal fistula or fissure before the Westminister Medical Society. in the course of which, after adverting to the causes, seat, and semeiology of that unfortunate accident, he recommends the use of a simple India rubber bottle as likely to afford relief. The aperture forming the communication between the vagina and neck of the bladder is occasionally large, and the intention in using the instrument, therefore, would be, that it should effectually form a partition or complete plug between these two organs, thus preventing the constant flow of urine through it, and the consequent great irritation, inflammation, and thickening of the callous edges.

The apparatus consists of a common India rubber bottle of moderate size, but free, if possible, from those lines and ornamental tracings which are placed on some, as they render them more liable to burst on distension, owing to the unequal thickness. It should be of a pyriform shape, without any shoulders or sudden bulging out from the neck or stalk of the bladder, as the latter form interferes with the ready extraction of the instruments when required, or at least occasions unnecessary pain in removing it. To the neck of the bottle is attached a mount containing a female screw, at the side of which is a small stop-cock. The other portion of the apparatus consists of a small condensing syringe, of the shape and size of the common breast-pump, the distal end of which terminates in a male screw, corresponding to the one attached to the bottle. The latter being well oiled or larded, and the air pressed out of it, is folded longitudinally, and carefully passed up into the vagina, until the lower end or mount is at the vulva. A certain portion of air is now drawn up into it by the simple removal of pressure, but it is not sufficiently distended till the condensing syringe is fixed, and a few strokes of the piston used. The number required must depend on circumstances, but will soon be known by the patient complaining that the further distension begins to give pain. The stop-cock is now turned, the syringe disengaged, and a napkin applied to the vulva, and fastened as usual.

The bottle is to be removed for a short time every evening, previously allowing the air to escape by the stop-cock, the vagina to be gently syringed out with warm water, and the bottle, after having been washed, to be replaced. Should its presence at first cause pain or inconvenience, it may be obviated by complete rest, the use of the hip-bath, and by warm vaginal injections. The comfort resulting from the use of the instrument is soon perceived, as it prevents that constant flooding of water which had previously taken place, excoriating the parts, and rendering the bed and clothes almost unfit for use. At the commencement of the treatment, it may be advantageous to pass the catheter occasionally, should there be any difficulty in passing urine by the natural passage. The daily removal of the instrument after passing urine, and the washing out of the vagina, will tend to allay inflammation, and prevent the accumulation of any irritating secretion about the affected parts. If possible it is better to have a second bottle, using it on alternate days, allowing the one removed to remain in water for a time, and then to be exposed to the air until again required. It will be rendered more durable by encasing it in an oil-skin covering, or by coating it with wax.

Three cases are narrated by Dr. Reid, in which the adoption of this apparatus afforded relief.—*Lancet*, Feb. 18, 1843.

---

### CAPACITY OF THE LUNGS.

At the Academy of Sciences, Paris, January 23, 1843, M. Bourgery read a memoir on the relation existing between the structure and fundamental capacity of the lungs in both sexes, and at various periods of life.

Experiments were made with a hydro-pneumatic apparatus on 70 persons (50 male, 20 female,) from which the following results were deduced:

The respiratory act, *cæteris paribus*, is more forcible in proportion to the youth and slender make of the individual. No condition of strength or health is capable of supplying the place of youth.

Respiration in the male is double the volume of that of the female for the same age. The maximum for both sexes occurs at the age of thirty years.

In a well-formed person of that age, forcible respiration represents the quantity of 2.50 to 1.30 litres for the male; and of 1.10 to 2.20 litres for the female; in the boy of fifteen years 2 litres; and in the old man of eighty 1.35 litres.* The volume of air required for ordinary respiration gradually increases with age. The ratios between the ages of seven, fifteen, twenty, and eighty, are geometric, and represented by the numbers 1, 2, 4, and 8. The well-formed adult respires habitually the quadruple of the young child, and the double of the female or child of fifteen years, while the old person respires the double of the adult. This progressive increase, or necessity for a greater volume of air, expresses the diminished power of the lung as an organ of hæmatosis; hence, the latter decreases from infancy to old age, in proportion to the following numbers: 1, $\frac{1}{2}$, $\frac{1}{4}$, and $\frac{1}{8}$.

In forced respiration the permeability of the lung to air presents two periods; one ascending from infancy to thirty years, the other descending from thirty to old age. The former increases in a regular ratio of 1, 2, and 3, from seven to fifteen and thirty years; the latter decreases from 3 to $2\frac{1}{2}$ between thirty and fifty years; and from $2\frac{1}{2}$ to $1\frac{1}{4}$ between fifty and eighty years of age.

Taken on the whole, the respiration is trebled within the space of twenty-three years in youth, and increases by 1-9th for each year. After manhood it diminishes by 2-5ths in twenty years, or by 1-100th for each year. From fifty to sixty years it decreases by 1-5th in ten years, or 1-50th for each year. And in old age, from sixty to eighty, it diminishes by nearly $\frac{1}{2}$ or 1-20th for each year. This gradual decline of the respiratory power must contribute in great measure to the gradual extinction of the powers of life as old age advances.

This latter proposition is further confirmed by the fact that the ratio of ordinary to forcible inspiration diminishes as the age advances. At seven years of age this ratio is as 1 to 12; at fifteen, as 1 to 10; at twenty, as 1 to 9; at twenty-five and thirty, as 1 to 6; at sixty, as 1 to 3; at eighty years of age, as 1o $\frac{1}{2}$ or $\frac{1}{8}$. Thus, the young man has in reserve for violent exertion an immense respiratory faculty, while the aged person is quickly "winded."

The respiratory faculty is gradually worn out by the laceration of the capillary aerian and sanguineous

---

* The *litre* is 1.700 of an English pint.

canals; this laceration occurs, in a greater or lesser degree, in all powerful respiratory efforts. It begins at an early period, and increases gradually to old age, as a simple consequence of repetition of the respiratory act. It is increased by all diseases of the lungs. In its most aggravated form, this state of the lung causes a circulation of imperfectly oxygenated blood, and reduces the decrepid octogenarian to the locular lung and imperfect respiration of the reptile.

### PECULIAR APPEARANCES IN CATARACT.

Dr. Jacob called the attention of the society to an appearance which presented itself in the eye of a person upon whom he lately operated for cataract, in the City of Dublin Hospital. The man, thirty-three years of age, was, he said, what is called amaurotic, or, in other words, his vision was very defective, even in the other eye, which was free from cataract, and, therefore, he was unwilling to operate, from a conviction that he had an unsound retina to deal with; but at the earnest solicitation of the patient he consented to let him have the chance which the experiment afforded. The cataract was lenticular, and, although more of an amber tint than is usual at this time of life, was otherwise not uncommon. The lens freely broken up with the needle through the cornea, and was easily separated into pulp and fragments, some of which fell into the anterior chamber, and no inflammation requiring attention followed. In a month the greater part was absorbed, and in six weeks the whole, leaving a shred of opaque capsule attached to the margin of the pupil, but not large enough to interrupt the passage of light. As the cataract, however, disappeared, the iris became studded with delicate brilliant scales of metallic lustre, so numerous and large as to be easily visible with the naked eye, and still more conspicuous with the assistance of a lens. They were irregular in form, but with surfaces so plane and polished that they reflected the light freely, resembling, in a remarkable manner, the particles of mica in granite. The appearance continued until the man was discharged, having been visible for about a month, and may probably continue so for some time. Sight, as had been predicted, was not restored, the retina being unsound. Dr. Jacob reminded the society that earthy, and perhaps crystalline, deposits in the lens and its capsule were not very uncommon, and that they had been met of so dense a nature as to lead to the application of the term ossification to them, although not to be considered at all of the nature of real bone. They are probably phosphate of lime, or perhaps ammonio-phosphate of magnesia with phosphate of lime, but that he left to the chemists to determine. He said that on another occasion, in breaking up a cataract of somewhat the same appearance, he was surprised to see a quantity of what appeared to be delicate needle-shaped crystals diffused among the fragments, but these disappeared with the cataract as it was dissolved.

He also exhibited a drawing of a capsular cataract, the consequence of injury, which he had removed successfully, and which had presented on the surface an appearance of such metallic lustre that he was obliged to make the artist represent it with silver leaf, and added that these brilliant cataracts, in a less marked form, were not very uncommon, but in all of them the disease was of long standing. Earthy deposits, he observed, were frequently found in the body of the lens in horses blind from cataract, consequent on inflammation. The shell of bone sometimes found within the choroid of disorganised eyes, and generally called ossified retina, he observed was probably of the same nature as these lenticular deposits. —*Trans. Surg. Society of Ireland, from Dub. Med. Press.*

### RELATIVE VALUE OF QUININE IN LARGE DOSES, AS A REMEDY IN TYPHUS.

A commission appointed to examine into the correctness of a memoir addressed by Broqua to the French Academy of Medicine, reports that some of the cases cited in the memoir are not proved to have been of a veritably typhoid character, and in others no proof is adduced of the quinine administered having been the means of cure. The report of the commission (if not belied by the journal which informs us of its presentation) sarcastically enough remarks, that " one interesting fact confirmed by Sig. Broqua's memoir is the *harmlessness* (l'innocuité presque constante) *of the sulphate of quinine* in large doses;" and it recommends that the memoir should be *honourably* shelved! In the discussion that followed its reading, M. Piorry stated, that in typhus fever, with engorgement of the spleen, he had seen quinine prove serviceable, which had not been the case when the fever was unaccompanied with splenic lesion. M. Martin Solon, who had employed the remedy under the personal inspection of Sig. Broqua at the Hôpital Beaujon, admitted that in cases in which the fever assumed a remittent type quinine was useful, but that remittent typhus was rare,—at least at Paris. "Of five severe cases of typhus fever, in which quinine had been given, death had resulted in three instances; and in the two others, recovery had only taken place after a considerable lapse of time, and without any evidence to show that the sulphate of quinine had been the means of hastening it. In the post-mortem examinations of the subjects who had died (says M. Martin Solon,) I failed to detect any peculiar alteration that I could fairly attribute to the large doses of the sulphate; it had passed in a manner imperceptibly through the stomach and intestines. A symptom I observed in those who recovered from the disease was a remarkable depression of the circulation. In short, I consider the advantage attributed to the sulphate to be more than doubtful." Much doubt was afterwards expressed by several members of the Academy as to the *innocuity* of large doses of quinia or its sulphate; but finally the terms of the report were adopted, and the memoir was shelved by a majority of voices.—*London Lancet,* Feb. 25, 1843. *From Gaz. des Hop.*

### TREATMENT OF TINEA FAVOSA.

The parasitical origin of this disease is regarded as demonstrated by the physicians of the General Hospital, Vienna. A division of this hospital is set apart for the treatment of chronic diseases of the skin, and the experiments there made in the treatment of tinea favosa seem to show that the local application of caustics (lunar caustic, caustic potass, &c.) is the only mode of treatment followed by beneficial results. Several cases were cured within two months by the local use of a saturated tincture of iodine.—*Provin. Med. Journal,* Feb. 4, 1843.

Blainville's definition of a ligament is "a tendon without muscles." His definition of a tendon was "a ligament, with a muscle attached."—*London Lancet.*

